# A Computational Fluid Dynamics Investigation of a Flapping Hydrofoil as a Thruster

**DOI:** 10.3390/biomimetics8020135

**Published:** 2023-03-25

**Authors:** Luca Alberti, Emanuele Carnevali, Daniele Costa, Andrea Crivellini

**Affiliations:** Department of Industrial Engineering and Mathematical Sciences, Polytechnic Marche University, 60131 Ancona, Italy

**Keywords:** carangiform thruster, propulsive performance, computational fluid dynamics, NACA0015, flapping foil, discontinuous Galerkin, Spalart-Allmaras, dynamic stall

## Abstract

The paper features a computational fluid dynamics study of a flapping NACA0015 hydrofoil moving with a combination of sinusoidal heaving and pitching. Several kinematic configurations are explored, varying sequentially pitch and heave amplitude, Strouhal number and phase angle, in an attempt to determine the influence of each parameter on the propulsive performance. To optimize efficiency the angle of attack should assume the highest value that also avoids the arise of the leading edge vortex generated in the dynamic stall state. At low Strouhal number optimum is reached at high heave amplitudes, which correspond to the configurations minimizing the hysteresis in the $\left(C_{y},C_{x}\right)$ plane. The same outcome in terms of hysteresis minimization has been verified to occur when optimal phase shift was considered. Differently, when the Strouhal number and the angle of attack become higher, to exploit efficiently the lift increment owed to dynamic stall it emerged the necessity of adopting low heave amplitude to improve separation resistance, avoiding the occurrence of deep stall.

## 1. Introduction

In the field of autonomous underwater vehicles (AUVs), bio-inspired solutions have been sought in the last three decades as a source of improvement in terms of propulsive efficiency and maneuverability. As a matter of fact, fishes and marine mammals are faster and nimbler than their robotic counterparts: AUVs turnabout radius is normally a multiple of the robot hull length, namely from two to six times, whereas a biological swimmer is capable to reverse its course without breaking, with a radius of curvature of the order of one third of its body length. The cost of transport, which measures the energy spent to cruise at a given speed, is also significantly lower for aquatic animals when compared to the state-of-the-art of modern nautical technology [1]. Therefore, several prototypes of swimming robots have been manufactured by researchers worldwide in the last thirty years, and an extensive review is provided in [2].

Despite the efforts made to pursue the considerable potential payoffs of marine animals’ locomotion, the performance of biological systems are still far to reach. Indeed, the possibility to emulate effectively the swimming modes developed by aquatic animals over thousands of years of evolution depends from the understanding of the fluid mechanics principles of swimming locomotion. In order to address this very ambitious objective, computational fluid dynamics (CFD) analysis represents an invaluable tool to investigate the propulsive performances of biological and bio-inspired thrusters.

According to swim mechanics, thrust force originates from the momentum transfer due to the interaction between the fish body and the surrounding water [3]. Particularly, body and caudal fin (BCF) swimmers generate thrust by bending their tails and caudal fins following specific undulation patterns. BCF locomotion is further expanded in five swimming modes characterized by the percentage of the body involved in the tail undulations. Thus, carangiform and subcarangiform swimmers generate thrust by bending respectively half and the last third of their tails. The motion law commonly adopted by biologists and roboticists to model the shape of the tail as a function of time is Lighthill’s travelling wave, an harmonic function whose amplitude increases moving towards the caudal fin [4]. On the other hand, in thunniform locomotion, thrust generation is mainly due to the caudal fin motion, where tail motion is mostly confined. Here, the fin traces an undulating path to adjust its angle of attack and prevent flow separation [5]. Indeed, thunniform locomotion is the most efficient swimming mode in BCF locomotion.

As stated before, several prototypes of bio-inspired underwater robots propelled both by carangiform and thunniform locomotion have been designed by researchers in the last thirty years. In the literature, the most common solution adopted to manufacture a carangiform swimming robot consists of a sealed forebody hinged to a piecewise flexible tail embodying a multi-joint, open-chain mechanism driven by dedicated servomotors or by a single rotary actuator [6,7,8]. A similar architecture has been employed to drive the caudal fins of thunniform swimming robots [9]. When a multi-joint mechanism is adopted to approximate the tail undulation patterns of the aforementioned swimming modes, it results easy to prove that the links of the system oscillate following an harmonic motion law. As a result, the caudal fin, which coincides with the tail linkage end effector, performs an harmonic roto-translation called flapping, where the individual components of the resulting motion are oscillation functions characterized by different amplitudes but the same frequency, and a constant phase shift. When a flapping caudal fin is employed as the thruster of a bio-inspired underwater robot, the quantification of its performance in terms of thrust generation and propulsive efficiency is a fundamental issue in the design process. As a matter of fact, in order to size the robot actuation system, the propulsive loads generated by the fin must be known as a function of the geometric and kinematic parameters of flapping motion.

To this end, this paper presents a detailed investigation of the dynamic performance of a roto-translating foil predicted by means of computational fluid dynamics. Here, the fin is simulated as a stand-alone thruster and the numerical predictions allow a complete characterization of its propulsive behavior. Aside from comparative considerations, the analysis results can be also exploited to compute the dynamics of a swimming robot and size its driving systems [8]. CFD analysis has been extensively employed in the field of biomimetics. In [10], the authors performed a two-dimensional analysis on the base ornithopter configuration of an insect flying robot using commercial CFD codes: the results have yielded deeper insights regarding the influence of varying flapping frequency on critical flow metrics regarding adequate lift and thrust generation. Flying systems have been investigated also in [11], where CFD methods have been employed to model the transitional aerodynamics of the variable camber morphing wing. Numerical simulations have been also exploited in the marine field to model the hydrodynamic performance of manta-like flapping [12].

Unlike other papers on the subject, the goal here is to provide a comprehensive performance-oriented manual on rigid flapping thrusters, which could be exploited as a design tool. Moreover, this work advances novel considerations on the physical effects of each kinematic parameter and proposes new perspectives on the cause-and-effect relationships between the wake structure and the propulsive performance, under a broad range of kinematic conditions. The heave amplitude, in particular, was varied in an interval that has seldom been studied in the past.

The paper is organized in the following way: Section 2 outlines the kinematics, introducing the fundamental expressions of the flapping motion and the parameters of interest. In Section 3 the numerical setup of the simulations is presented, including the spatial and temporal discretization schemes, the turbulence model implemented and the special treatment reserved to moving boundaries. The results are collected in Section 4, preceded by a brief description of the mesh and the definition of the main propulsive performance indicators. Finally, the most significant conclusions are drawn in Section 5.

## 2. Motion Kinematics

Fishes and aquatic mammals arrived to their current locomotion capabilities through a process of biological optimization, driven by natural selection. As a result, a wide and complex variety of swimming behaviors have developed, usually classified into (i) undulatory, (ii) oscillatory, (iii) pulsatile jet-based and (iv) drag-based motions. For a detailed explanation of each class the interested reader can refer to Smits’ well-known review [13]. The species adopting motion behaviors that fall within one or another of these categories further exhibit peculiar body shapes, with geometries and mass distributions that coupled with the adopted swimming configuration have the effect of optimizing their underwater motion.

A complete and exact representation of such a rich variety of configurations, if possible, would require the adoption of prohibitively complex models, rendering computational simulations unfeasible. However, it has been showed how for the oscillatory configurations, which include BCF swimmers, significant simplifications may be adopted and still derive a model retaining relevant information about the physics of the problem. For instance, various experimental investigations [5,14,15,16] showed that the approximation of the oscillatory regime through a flapping hydrofoil predicts the maximum efficiency in the same range of kinematic configurations found for cetaceans and carangiform fishes.

**Figure 1:**
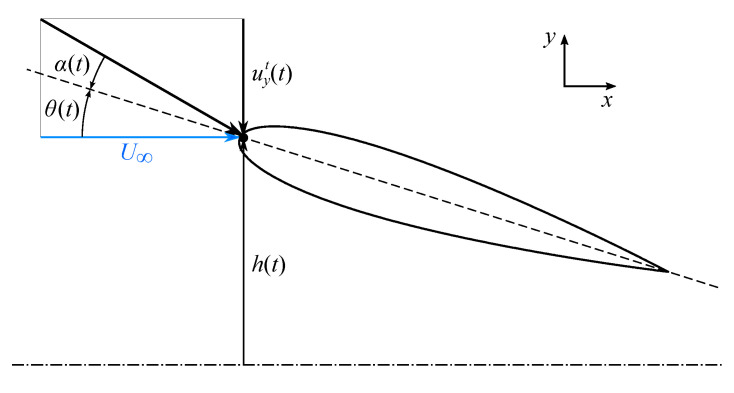
Scheme of the NACA0015 airfoil.

Within the flapping foil approximation, the body inertia contribution in the propulsion generation is discarded, with the motion assumed to be concentrated at the body end, namely at the propeller tail. The latter is then suitably approximated via hydrofoil profiles, moving according to kinematic laws approximating their natural motion. The analysis has here been restrained to a two-dimensional case, with the propeller approximated using a symmetric NACA0015 profile, whose schematic representation is given in Figure 1 together with some relevant kinematic parameters.

The profile is hence prescribed to move according to an harmonic law composed of a rotation around a pivotal point and a vertical translation, following the parametrization presented below:

(1) $$\begin{array}{c} \begin{array}{cc} \theta (t) & =\theta_{0}\sin\left(2\pi ft\right)\\ h(t) & =h_{0}\sin\left(2\pi ft+\psi \right)\;. \end{array} \end{array}$$

According to Equation (1), pitch amplitude and vertical displacement follow a sinusoidal variation with frequency *f*. The pitching motion occurs around a fixed center of rotation, with the maximum angular displacement given by $\theta_{0}$. The simultaneous heaving motion is characterized by an amplitude $h_{0}$ and present, with respect to the periodic rotation, a constant phase shift ψ.

From the above relations, the vertical and rotational velocities may be read as:

(2) $$\begin{array}{c} \begin{array}{cc} \omega_{z}^{t}\left(t\right) & =2\pi f\theta_{0}\cos\left(2\pi ft\right)\\ u_{y}^{t}\left(t\right) & =2\pi fh_{0}\cos\left(2\pi ft+\psi \right)\;, \end{array} \end{array}$$

where the superscript ‘*t*’ identifies the components representing the relative motion between the inertial and the moving reference frames. The velocity induced by the profile vertical motion affects the instantaneous, effective angle of attack, that according to the convention exposed in Figure 1 may be expressed as follows:

(3) $$\alpha \left(t\right)=\theta \left(t\right)+\arctan\left(\frac{u_{y}^{t}\left(t\right)}{U_{\infty }}\right)\;,$$

with $U_{\infty }$ being the horizontal free-stream velocity. The maximum value reached by α in the harmonic period is indicated by $\alpha_{max}$.

A global description of the profile kinematics widely adopted in the topic of flapping hydrofoils is provided via the Strouhal number

(4) $$St=f\frac{A}{U_{\infty }}\approx f\;\frac{2h_{0}}{U_{\infty }}.$$

This non-dimensional parameter groups together the profile oscillation frequency with a length scale that best characterizes the flow. In the context of flapping foils the characteristic length may be associated to the wake width *A*, here approximated by the total heave amplitude $2h_{0}$.

The predominance of heaving or pitching in the foil motion can be quantified by means of the dimensionless heave ratio, defined by Akoz et al. [17] as

(5) $$h^{*}=\frac{2h(t^{*})}{A_{TE}},$$

where $t^{*}$ is the time instant at which the trailing edge (TE) reaches its highest vertical position and $A_{TE}$ is the total vertical excursion of the TE. The motion is heave-dominated when $h^{*}>0.5$, while $h^{*}<0.5$ identifies a pitch-dominated kinematics.

In order to provide a comprehensive analysis of the propulsive performance related to the flapping motion, a wide range of kinematic configurations have been explored. Thrust and efficiency have been computed for several combinations of St, $\theta_{0}$, $h_{0}$ and ψ, which have been extensively recognized as the major kinematic factors influencing the propulsive capabilities of the foil.

## 3. Simulation Setup

As far as the numerical framework is concerned, the simulations were carried out adopting an incompressible, high-order discontinuous Galerkin (DG) solver. This DG solver was extensively validated over the years for a broad variety of fluid dynamics applications [18,19,20,21]. The current implementation with a moving reference frame was validated in [22] for a flow past a transversely oscillating cylinder, where the numerical resuts were compared with well established reference data.

The problem has been modelled adopting as governing equations the fully turbulent Unsteady Reynolds-Averaged Navier-Stokes equations (URANS), equipped with the Spalart-Allmaras [23] one equation model for the turbulent viscosity-like variable $\tilde{\nu }$. Such choice has been motivated by the widely demonstrated efficacy of this particular one-equation model in aerodynamic flow predictions, also showed in the framework of oscillating foil, reported for example in [24,25]. The validity of the Spalart-Allmaras turbulence model to simulate oscillating foils in a DG environment was addressed in a recent paper [26], where the numerical results showed good agreement with the experimental data available in literature.

To avoid dealing with moving meshes a relative reference frame, fixed with the NACA profile, is introduced. The governing equations are then formulated expressing the velocity components $u_{i}$ defined in the absolute (inertial) frame described in terms of the coordinates in the moving frame [27,28]. The relative motion between the two frames is then represented by an inter-frame velocity vector $U_{i}\left(t\right)=u_{i}^{t}\left(t\right)+\varepsilon_{ijk}{\omega_{j}}^{t}\left(x_{k}-x_{0,k}\right)$. The vectors $u_{i}^{t}$ and ${\omega_{j}}^{t}$, introduced in Equation (2), are now to be intended as expressed in terms of the foil-fixed relative frame coordinates, with $\left(x_{k}-x_{0,k}\right)$ representing the distance vector from the rotation point and $\varepsilon_{ijk}$ being the Levi-Civita tensor. In this way it is easy to see that $U_{i}\left(t\right)$ exactly identifies the profile velocity, as seen from the inertial frame, projected onto the relative one.

Exploiting the solenoidality of the velocity field $U_{i}$, the equations of motion assume the following form:

(6) $$\begin{array}{c} \begin{array}{cc} \; & \frac{\partial u_{i}}{\partial x_{i}}=0\;,\\ \; & \frac{\partial u_{i}}{\partial t}+\frac{\partial u_{i}u_{j}}{\partial x_{j}}=-\frac{\partial p}{\partial x_{i}}+\frac{\partial }{\partial x_{j}}\left(\left(\nu +\nu_{t}\right)\frac{\partial u_{i}}{\partial x_{j}}\right)-\varepsilon_{ijk}\omega_{j}^{t}u_{k}+U_{j}\frac{\partial u_{i}}{\partial x_{j}}\;,\\ \; & \frac{\partial \tilde{\nu }}{\partial t}+\frac{\partial \tilde{\nu }u_{j}}{\partial x_{j}}=C_{b1}\tilde{S}\tilde{\nu }+\frac{C_{b2}}{\sigma }\frac{\partial \tilde{\nu }}{\partial x_{j}}\frac{\partial \tilde{\nu }}{\partial x_{j}}-C_{w1}f_{w}{\left(\frac{\tilde{\nu }}{d}\right)}^{2}+\frac{1}{\sigma }\frac{\partial }{\partial x_{j}}\left(\left(\nu +\nu_{t}\right)\frac{\partial \tilde{\nu }}{\partial x_{j}}\right)+U_{j}\frac{\partial \tilde{\nu }}{\partial x_{j}}\;, \end{array} \end{array}$$

where the working variable for the pressure is to be intended as the specific pressure p=P/ρ and *d* is the minimum distance from the wall. The effective turbulent viscosity is derived as $\nu_{t}=f_{v1}\tilde{\nu }$, with $f_{v1}$, as well as $f_{w}$ and $\tilde{S}$, being a closure function that, together with the constants σ, $C_{b1}$, $C_{b2}$ and $C_{w1}$, defines the Spalart-Allmaras model. The interested reader may find a comprehensive description of the previously cited terms in [23].

The numerical scheme is constructed starting from a suitable partition of the physical domain Ω, where the exact solution of the above model lives, into a set of $n_{e}$ non overlapping elements $\Omega_{k}$:

(7) $$\Omega \approx \Omega_{h}=\overset{n_{e}}{\underset{k=1}{⋃}}\Omega_{k}.$$

The DG discretization is then obtained by considering an element based, modal expansion for the solution vector making use of base functions made up by hierarchical, orthonormal polynomials up to order *n*. The analytical solution is hence locally approximated via a finite-dimensional, piecewise-continuous polynomial, possibly discontinuous across element interfaces.

For the sake of compactness the state variables are collected in a solution vector $q={\left\{p,u_{i},\tilde{\nu }\right\}}^{T}$ so that the equations above may be represented in the following matrix notation:

(8) $$I^{0}\frac{\partial q}{\partial t}+\nabla \cdot \left(F_{c}+F_{d}\right)+g=0.$$

Here the term $I^{0}=I-I^{\left(1,1\right)}$, where $I^{\left(i,j\right)}$ denotes a single entry matrix, has been introduced to cancel the time derivative of pressure in the continuity equation, keeping the model consistent with the representation provided in Equation (6). Finally, $F_{c}$ and $F_{d}$ identify the convective and diffusive flux tensors, both function of time and system state, while the vector g, introduced to collect the source terms, contains the contributions of the non-inertial components arising from the inter-frame formulation.

In this way, the modal Galerkin expansion for the vector q reads:

(9) $$q_{k}=\sum_{i=1}^{m}\phi_{i}^{k}\left(x\right)\;Q_{i}^{k}\left(t\right)\;,$$

where m=(n+1)(n+2)/2 defines the number of degrees of freedom, the function $\phi_{i}$ identifies the *i*-th component of the polynomial base, and $Q_{i}$ collects the corresponding degrees of freedom of the solution vector. The index *k*, instead, takes into account the element-based character of the method, with the base functions $\phi_{i}$ having compact support.

Having a local approximations of the solution vector living in the polynomial space $P^{n}$ ensures a space accuracy of order n+1 for the velocity and eddy viscosity-like, and order of accuracy *n* for the pressure.

The numerical scheme is then constructed passing through the variational formulation of the above system of partial differential equations. Defining a test function v, living in the same finite-dimensional vector space of the solution vector, the weak formulation is obtained imposing an orthogonality condition among v and the governing equations and integrating by parts:

(10) $$\int_{\Omega_{k}}v\cdot \left(I^{0}\frac{\partial q}{\partial t}-\nabla v\cdot \left(F_{c}+F_{d}\right)+v\cdot g\right)d\Omega +\oint_{\partial \Omega_{k}}v\cdot \left(H_{c}+H_{d}\right)d\sigma =0,$$

where the element index, *k*, in the integrand functions has been dropped.

One of the key features of a DG method consists in the evaluation of a uniquely defined, normal, numerical fluxes $H_{c,d}$, depending on the adjacent elemental states and gradients. The convective flux, $H_{c}$, is here treated using the artificial compressibility flux method reported in [29], while for the viscous contribution, $H_{d}$, the well-known BR2 discretization scheme of Bassi and Rebay [30] is implemented.

Introducing in Equation (10) the previously defined *n*-th order modal expansion for q finally yields a system of linear ordinary differential equations (ODEs) for the global degrees of freedom (d.o.f.) vector **Q**:

(11) $$M^{0}\frac{dQ}{dt}+R=0\;,$$

where $M^{0}$ is a modified global, block diagonal mass matrix, characterized by null entries at the pressure degrees of freedom, Q is the global solution vector containing the solution d.o.f. and R is the residual vector, containing the surface and volume contribution of flux tensors and non−inertial source terms.

The system is discretized in time using a three-stages, third-order accurate Rosenbrock-type Runge-Kutta scheme ROS3P [31]. The latter is of linearly-implicit type, it allows the construction of the new time solution Q by freezing the Jacobian matrix ∂R/∂Q computation at each time step, avoiding hence any non-linear resolution procedure. The linear system arising from time discretization of Equation (11) is finally resolved using a flexible generalized minimal residual (GMRES) solver preconditioned by a highly parallel efficient *p*-multigrid algorithm, described in [32]. It should be underlined that due to the inter-frame velocity formulation, the boundary condition are non-homogeneous and the system is in turn non-autonomous, with a d.o.f. time dynamics that results to be function of not only the state vector but also, explicitly, of time.

## 4. Results

This section gives a brief description of the computational grid and introduces the main results in terms of flow fields and propulsive performance plots. The latter have to be intended as an integration of what was partially presented in the CFD analysis contained within the multiphysics study performed by Costa et al. [8].

**Figure 2:**
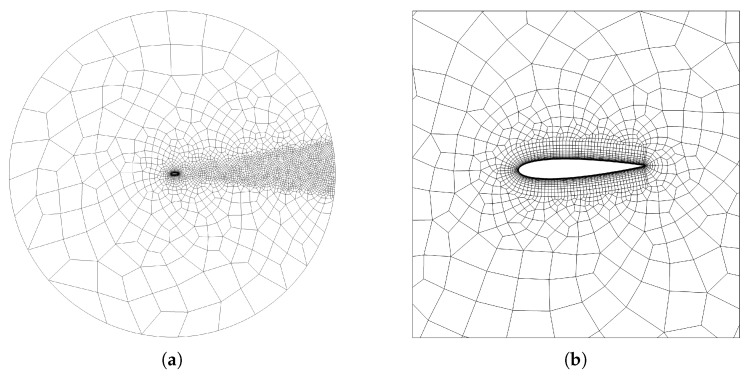
(**a**) Global mesh; (**b**) Close-up view of NACA0015.

The fluid domain is a circle of radius 25c, where *c* is the foil chord length, discretized by means of an unstructured mesh with $n_{e}=4670$ quadrangular elements, as illustrated in Figure 2a. The Open Source software employed for the mesh generation is GMSH [33]. The cells nearby the boundary of the profile are structured-like and adequately curved so to wrap around the foil curvature, as shown in Figure 2b. The face of these cells is approximated using a piecewise third order polynomial. The cells in contact with the profile have a height $y^{+}\approx 5$ and get progressively inflated outwards for a total of 19 layers. The region behind the foil is refined to enable an accurate study of the wake, which is directly correlated to the propulsive features of the flapping motion. In particular, it is well known that the forward motion of a flapping foil is characterized by counter-rotating eddies forming a jet-like wake, typically denoted as reverse Von Kármán vortex street [34]. In order to verify the independence of the results upon the mesh we did not follow a grid refinement procedure, typical of low-order methods, but rather a check increasing the polynomial degree of the solver, as done in [26], which is known to be a viable alternative for high-order schemes. Simulations were carried out up to n=6.

**Figure 3:**
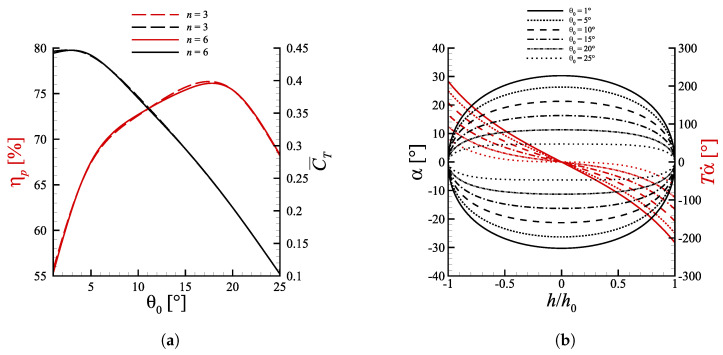
(**a**) Propulsive efficiency and mean thrust coefficient as a function of the pitch amplitude for cases with $\psi =90^{\circ }$ and St=0.1935. The nearly overlapping curves at polynomial degree n=3 and n=6 indicate that convergence is reached for n≥3. (**b**) Spatial orbits of α and $T\dot{\alpha }$. Lines with the same style share the same $\theta_{0}$.

The reason was just to ensure the convergence of the results, as a matter of fact the propulsive performances remained practically the same once n=3 was reached, see for example the performance data reported in Figure 3a. The time integration was done with a step size ranging from $0.001c/U_{\infty }$ to $0.05c/U_{\infty }$. The choice was driven by the need to obtain a satisfactory trade-off between numerical stability and computational expense.

For the sake of generality, the forces in *x* and *y*-direction, i.e., $F_{x}$ and $F_{y}$, and the torque $M_{z}$ exerted on the foil are normalized, ending up with the drag, lift and moment coefficients below

(12) $$C_{x}=\frac{F_{x}}{1/2\;\rho \;b\;c\;U_{\infty }^{2}}\;,\;C_{y}=\frac{F_{y}}{1/2\;\rho \;b\;c\;U_{\infty }^{2}}\;,\;C_{M}=\frac{M_{z}}{1/2\;\rho \;b\;c^{2}\;U_{\infty }^{2}}\;,$$

where ρ is the fluid density and *b* is the (unit) span. It should be noted that the force components $F_{x}$ and $F_{y}$, together with the torque $M_{z}$, are to be intended as expressed in the absolute reference frame, meaning that the referred *x* and *y*-direction coincide with the two coordinate axis of the inertial frame.

The propulsive performance associated to the oscillating motion is evaluated in terms of mean thrust coefficient ${\bar{C}}_{T}=-{\bar{C}}_{x}$ and propulsive efficiency $\eta_{p}$. Essentially, the assessment is based on the amount of propelling force that the foil is able to produce per unit period and on the effectiveness of this production mechanism in relation to the power spent to activate the body. In mathematical form the two quantities can be defined as

(13) $$\begin{array}{c} {\bar{C}}_{T}=\frac{1}{n_{c}T}\int_{0}^{n_{c}T}C_{T}\;dt \end{array}$$

(14) $$\begin{array}{c} \eta_{p}=\frac{{\bar{C}}_{T}}{{\bar{C}}_{P}}\;, \end{array}$$

where ${\bar{C}}_{P}$ is the mean power coefficient

(15) $${\bar{C}}_{P}=-\frac{1}{n_{c}T}\int_{0}^{n_{c}T}\left[C_{y}\frac{u_{y}^{t}}{U_{\infty }}+C_{M}\;\frac{c\omega_{z}^{t}}{U_{\infty }}\right]\;dt\;,$$

while $n_{c}$ and *T* represent the number of cycles selected for the average and the period, respectively.

**Figure 4:**
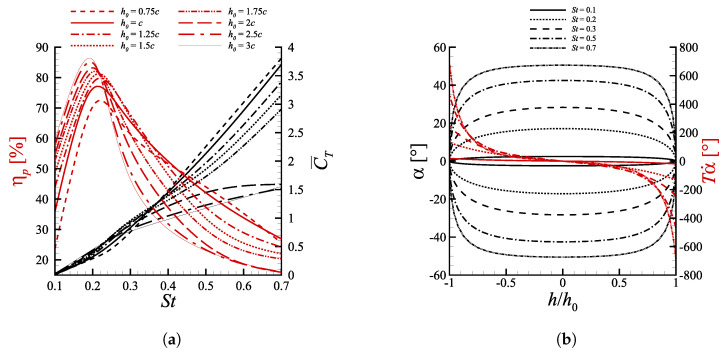
(**a**) Propulsive efficiency and mean thrust coefficient as a function of the Strouhal number for cases with $\psi =90^{\circ }$ and $\theta_{0}=15^{\circ }$. Lines with the same style share the same $h_{0}$. (**b**) Spatial orbits of α and $T\dot{\alpha }$. Lines with the same style share the same St.

**Figure 5:**
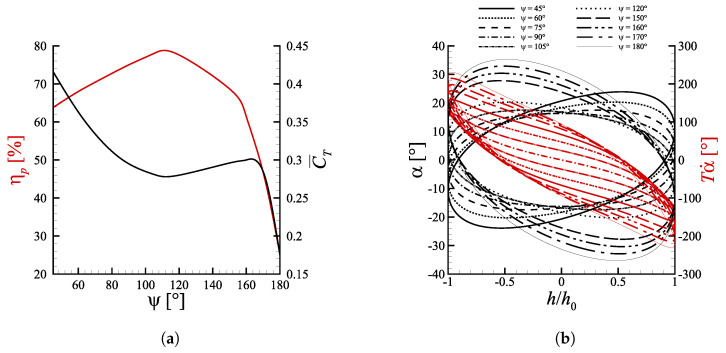
(**a**) Propulsive efficiency and mean thrust coefficient as a function of the phase shift for cases with St=0.1935 and $\theta_{0}=15^{\circ }$. (**b**) Spatial orbits of α and $T\dot{\alpha }$. Lines with the same style share the same ψ.

**Figure 6:**
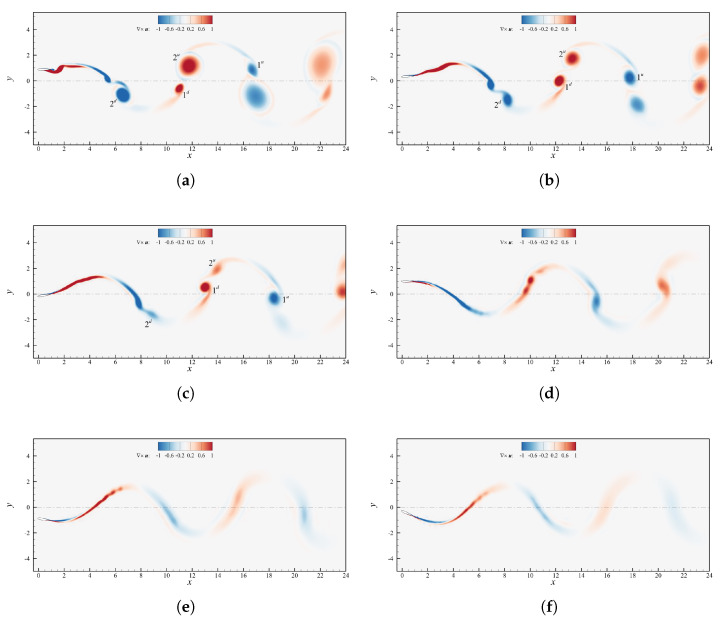
Contour plot of the instantaneous vorticity field for case $h_{0}=c,\;\psi =90^{\circ }$ and St=0.1935. (**a**) $\theta_{0}=1^{\circ }$; (**b**) $\theta_{0}=5^{\circ }$; (**c**) $\theta_{0}=10^{\circ }$; (**d**) $\theta_{0}=15^{\circ }$; (**e**) $\theta_{0}=20^{\circ }$; (**f**) $\theta_{0}=25^{\circ }$.

**Figure 7:**
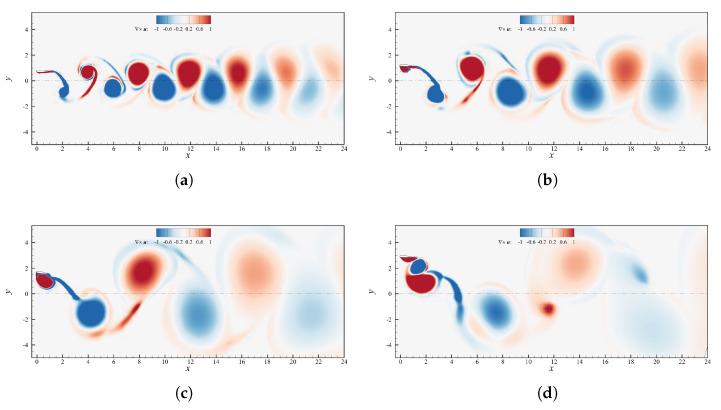
Contour plot of the instantaneous vorticity field for cases with $St=0.5,\;\psi =90^{\circ }$ and $\theta_{0}=15^{\circ }$. (**a**) $h_{0}=0.75c$; (**b**) $h_{0}=1.25c$; (**c**) $h_{0}=2c$; (**d**) $h_{0}=3c$.

**Figure 8:**
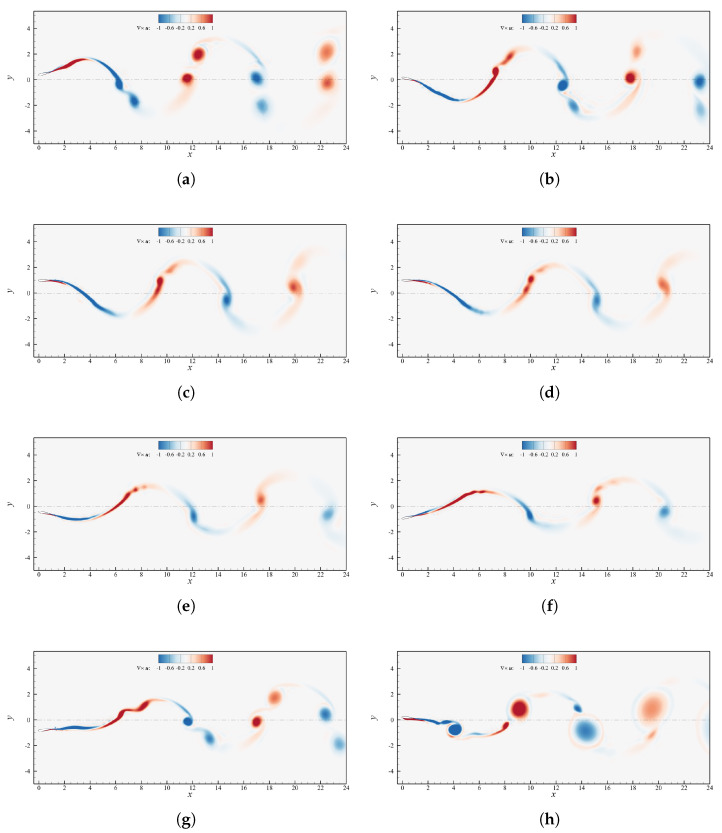
Contour plot of the instantaneous vorticity field for case $h_{0}=c,\;\theta_{0}=15^{\circ }$ and St=0.1935. (**a**) $\psi =45^{\circ }$; (**b**) $\psi =60^{\circ }$; (**c**) $\psi =75^{\circ }$; (**d**) $\psi =90^{\circ }$; (**e**) $\psi =105^{\circ }$; (**f**) $\psi =120^{\circ }$; (**g**) $\psi =150^{\circ }$; (**h**) $\psi =170^{\circ }$.

All the kinematic configurations addressed in this paper are featured by a Reynolds number $Re=U_{\infty }c/\nu =300,000$. As reported in [26] this value is enough to employ a fully turbulent model, as for flapping foils the latter provides results analogous to those obtained using an algebraic transition model. This holds true as long as there is no combination of extremely small St and high α, which is not present in any of the simulated cases. The center of rotation of the foil is located inside the profile, along the chord line, at 1/17c from the leading edge. Since the aim was to analyze the sensitivity of the propulsive performance to the kinematic variables discussed in Section 2, a series of tests were performed by changing just one parameter and keeping all the others constant. The choice of the test cases was made starting from a known efficient flapping configuration typical of BCF swimmers, characterized by $\theta_{0}=15^{\circ }$, $h_{0}/c=1$, $\psi =90^{\circ }$ and St=0.2. The kinematic parameters in play were then varied between lower and higher values for a reasonably extended interval, in order to include a representative number of operational conditions. Table 1 collects the complete list of cases and the resulting propulsive efficiency and mean thrust coefficient. The graphical version is given by Figure 3, Figure 4 and Figure 5. Moreover, Figure 6, Figure 7 and Figure 8 depict the vorticity fields of the majority of the cases contained in Table 1. Indeed, despite someone remarks that there is no one-to-one connection between the wake morphology and the propulsive performance [13], the visualization of the vortical structures surely helps in understanding the physical mechanisms that improve thrust and/or efficiency. Some of the kinematic configurations have been also animated over few cycles, in order to study the shedding of the vortices during upstroke and downstroke with greater detail. These animations are provided as Supplementary Materials.

In the following paragraphs the effect of each kinematic parameter is isolated and dissected.

### 4.1. Angle of Attack and Dynamic Stall

**Figure 9:**
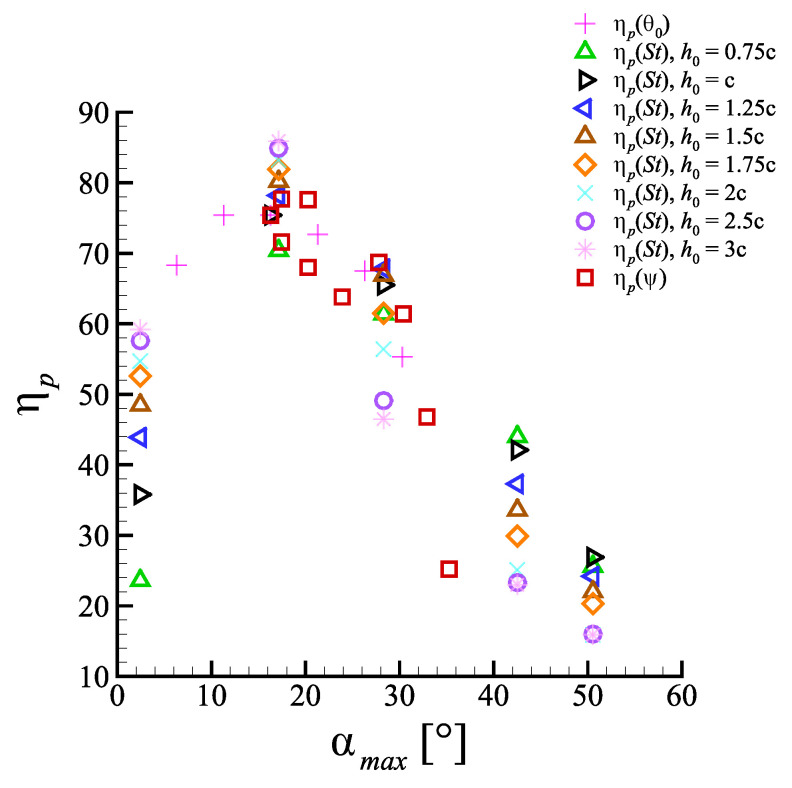
Propulsive efficiency as a function of the peak angle of attack.

Numerous works recognized the angle of attack as a crucial parameter to control the propulsive performance of a flapping foil [15,35,36]. Figure 9 presents a compact portrait of the entire space of configurations simulated, plotted in terms of propulsive efficiency against maximum angle of attack.

Even though efficiency is affected by the interplay of all kinematic parameters, a global optimum is always identifiable, for the explored configurations, in correspondence of those generating $\alpha_{max}$ between $10^{\circ }$ and $25^{\circ }$. This is in fair accordance with what reported in [36], but even so high values of efficiency are still found for $\alpha_{max}$ values falling well above the static stall condition.

Due to the circulation-based aspects of the considered propulsion mechanism, the persistence of high efficiency at elevated $\alpha_{max}$ suggests the presence of a continuous production of lift that might be traced back to the onset of dynamic stall process [37,38]. Dynamic stall is a complex phenomenon affecting airfoils in conditions of unsteady motion. It determines a delay in severe separation, enabling the profile to produce lift up to angles of attack well above the static, stall-inducing value. Within the considered framework of flapping kinematics, the contribution coming from the aforementioned unsteady motion manifests as a time-dependent rate of change in the angle of attack $\dot{\alpha }=d\alpha /dt$. With its shape and magnitude intimately dependent upon the tested motion parameters, such quantity proved to be of fundamental relevance, being involved, in particular, in the control of some of the dynamic stall aspects critically affecting the profile propulsive performances, see Section 4.3.

As the dynamic stall regime sets in, imposing a dynamic delay of stall-like separation, a lift increasing tendency along with α is observed. This follows a rather complex behavior and is eventually characterized, for the larger values of α, by sudden increments owed to the arise of local separation phenomena and the subsequent vorticity organization at the solid boundary. These coherent structures, arising over the suction surface of the foil, are usually referred to as leading edge vortex (LEV), or also as dynamic stall vortex (DSV) in the specialized literature [39,40,41]. Despite the significant lift increase that such structures have been demonstrated to provide, the obtained results seem to suggest how the optimal condition, from an efficiency viewpoint, is identified by configurations limiting, when not avoiding, their formation. Following the observations reported in [39], the above assertion may be rephrased by saying that for a configuration producing large thrust efficiently, $\alpha_{max}$ should be in the so-called linear region of the $\left(C_{L},\;\alpha \right)$ plane, found within the pre-stall regime.

In other words, analogously to steady aerodynamics, the peak angle of attack should be kept low enough so to avoid massive separation if one wants to produce thrust in an efficient way.

The authors are aware of the limitations related to the application of turbulence models to unsteady aerodynamic problems, particularly when accurate capturing of loads at large α and the onset of boundary layer separation are required. Nonetheless, the framework of URANS simulations, employing several closure equations among which the SA version used in this paper, proved its capability of yielding results qualitatively significant when investigating dynamic stall [40].

**Figure 10:**
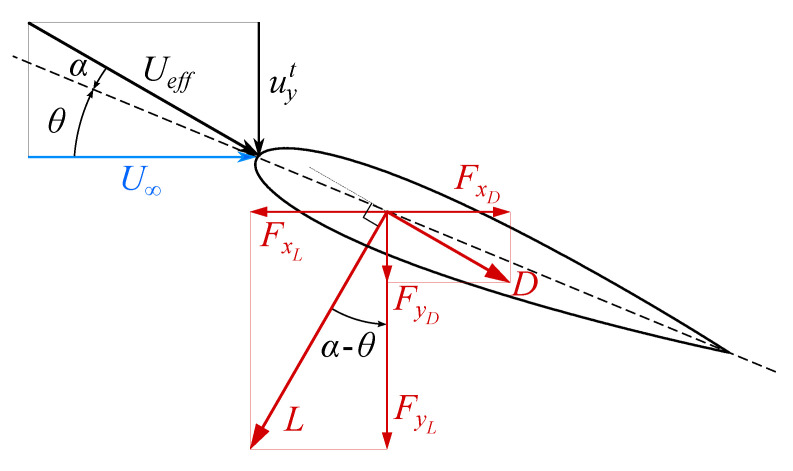
Lift and drag vector decomposition into streamwise and vertical contributions.

For optimal conditions $\alpha_{max}$ should not be too small either. To clarify why one can refer to Figure 10 and consider the following argument. For heave-dominated motions the power produced by the torque $M_{z}c\omega_{z}^{t}$ has been observed to be much smaller than the one generated by thrust and drag. Indeed, in the computations reported here, its contribution to ${\bar{C}}_{P}$ is less than 2% and hence it can be neglected. With this hypothesis the instantaneous output and input power $P_{out}$, $P_{in}$ are given by

(16) $$\begin{array}{cc} P_{out} & =\left(\underset{F_{x_{L}}}{\underset{︸}{L\sin\gamma }}-\underset{F_{x_{D}}}{\underset{︸}{D\cos\gamma }}\right)U_{\infty }\;, \end{array}$$

(17) $$\begin{array}{cc} P_{in} & =\left(\underset{F_{y_{L}}}{\underset{︸}{L\cos\gamma }}+\underset{F_{y_{D}}}{\underset{︸}{D\sin\gamma }}\right)u_{y}^{t}\;, \end{array}$$

with γ=α−θ. The propulsive efficiency is then computed as

(18) $$\eta_{p}=\frac{\int_{0}^{n_{c}T}P_{out}\;dt}{\int_{0}^{n_{c}T}P_{in}\;dt}=\frac{\int_{0}^{n_{c}T}\left(L\sin\gamma -D\cos\gamma \right)U_{\infty }\;dt}{\int_{0}^{n_{c}T}\left(L\cos\gamma +D\sin\gamma \right)u_{y}^{t}\;dt}\;.$$

From Equation (18) it is easy to verify that $\eta_{p}\approx 1$ when L≫D, due to the fact that

(19) $$\frac{u_{y}^{t}}{U_{\infty }}=\pi St\cos\left(\frac{\pi \;St\;U_{\infty }}{h_{0}}t\;+\psi \right)=\tan\gamma \;.$$

This means that as long as lift dominates drag, the efficiency is close to the unit regardless of the modulus of the lift and its orientation. Otherwise the efficiency starts to decay along with the lift-to-drag (L/D) ratio, due to the progressive emergence of the detrimental contribution of drag both in the streamwise and vertical directions; this comes as no surprise since the L/D ratio represents the aerodynamic efficiency of a body. In this context the dependence of $\eta_{p}$ on γ, and thus partially on St, even if it exists, is much less impactful. Since prior stall one can imagine that, for a symmetric profile in quasi-static conditions, $L\propto \alpha U_{eff}^{2}$, at very small angles of attack the lift is almost absent whereas the drag is not, being $D\propto \left(C_{D_{0}}+\alpha^{2}\right)U_{eff}^{2}$ with $C_{D_{0}}>0$. This leads to a very small L/D ratio which determines not only a very small ${\bar{C}}_{T}$, but also an unsatisfactory $\eta_{p}$.

### 4.2. Pitch Amplitude

The pitch amplitude is also an important quantity, because it does not only set the maximum excursion of the pitch angle, but it also modulates the pitch rate. Figure 3a displays the performance sensitivity to the pitch amplitude $\theta_{0}$, maintaining St=0.1935, $h_{0}=c$ and $\psi =90^{\circ }$. The pitch amplitude was varied using the stencil $\left[1^{\circ },5^{\circ },10^{\circ },15^{\circ },20^{\circ },25^{\circ }\right]$. Note that the St was set to such value in order to get $St_{TE}=0.2$ for the reference case $h_{0}=c$, $\theta_{0}=15^{\circ }$, $\psi =90^{\circ }$, where $St_{TE}=fA_{TE}/U_{\infty }$ may be thought of as the ‘real’ Strouhal number based on the TE total excursion.

The thrust coefficient presents a global maximum presumably within the interval $1^{\circ }<\theta_{0}<5^{\circ }$ and quickly drops for greater values of total heave excursion. This is due to the progressive decrease of $\alpha_{max}$ as $\theta_{0}$ is increased, which in the end brings the profile in a situation where lift is practically zero. The fact that the maximum mean thrust is produced at peak angles of attack over $26^{\circ }$ may be explained again with the dynamic stall phenomenon, whose impact is amplified at low pitch amplitudes due to the higher rate of change of the angle of attack, as displayed by Figure 3b. The maximum efficiency has a value of approximately 76% around $15^{\circ }<\theta_{0}<20^{\circ }$. To understand why efficiency is maximized in this region, one can recall what was said in Section 4.1 about the dependence of $\eta_{p}$ on the L/D ratio. The interval $15^{\circ }<\theta_{0}<20^{\circ }$ is indeed where the ratio between lift and drag returns the highest value. For lower pitch amplitudes lift increases due to the dynamic stall effect, but drag increases even more and gives a lower L/D ratio. At higher pitch amplitudes, instead, the peak angle of attack is so small that lift is almost null, differently from drag which is non-zero even when α=0.

Figure 6 depicts the vorticity fields at varying pitch amplitude. Starting from Figure 6a we can see a typical 2P wake, in accordance with the nomenclature introduced by Roshko and Williamson [42]. The counter-rotating vortex pairs shed each half cycle are not balanced though, as the second vortices generated during upstroke/downstroke (indicated with $2^{u}$/$2^{d}$ in Figure 6a) are much bigger than the corresponding first ones (identified by $1^{u}$/$1^{d}$). As $\theta_{0}$ increases, the second vortices lose strength and at $\theta_{0}=10^{\circ }$ they become less intense than their counterparts, which seem to maintain instead the same size and level of vorticity (Figure 6b,c). Further increasing $\theta_{0}$ the second vortices start to be fragmented in multiple co-rotating swirling structures and become so weak that they merge almost immediately with the first vortices, leading to a sort of 2S wake which resembles a reversed von Kármán street. Above $\theta_{0}=20^{\circ }$ the instantaneous angle of attack always remains well below the value of static stall, therefore clear separation cannot be appreciated and no vortex gets formed downstream the foil, with the wake being basically streamlined. An interesting insight could be extracted by noting that the vortex pairs observed up to $\theta_{0}=15^{\circ }$ seem to be formed in two distinct phases. The first vortices are created at the extremes of the heave motion, where $\dot{\alpha }$ is maximum, and detach when the foil is approximately in the neutral position, meanwhile the second eddies arise past the centerline, where α reaches its maximum value, and detach when the profile approaches the point of maximum vertical displacement. This could suggest that $2^{u}$, $2^{d}$ are linked to the separation owed to the reaching of an angle of attack greater than the stall value, whereas $1^{u}$, $1^{d}$ are caused by the sudden change of α rather than its instantaneous value, as it can be appreciated in Figure 3b. On the base of this assumption $1^{u}$, $1^{d}$ would be nothing but the so-called starting vortices largely examined in literature [39]. Since the second vortices origin on the side of the foil opposite to where the profile is moving (lower surface in upstroke and upper surface during downstroke) their effect is beneficial in terms of thrust production, because they rotate contrarily to a classical von Kármán street. On the other hand, the first vortices rotate in the opposite sense, and so their contribution is detrimental or beneficial from a propulsive point of view, depending on the timing of the detachment. Specifically, if $1^{u}$ ($1^{d}$) detaches below (above) the certerline it most likely has a positive effect on thrust generation, otherwise it acts in drag favor. The positive or negative effect of the vortices depends indeed not only on the sense of rotation, but also on the position with respect to the neutral axis (see Section 4.4 for a more detailed motivation).

The fact that for small pitch amplitudes α is larger, especially around h=0, produces stronger second vortices and allows to obtain high ${\bar{C}}_{T}$. This, in turn, helps to maintain efficiency at discrete levels. On the base of these reasonings one could argue that the efficiency is decently high even when the second vortices weaken as in Figure 6c, despite the first vortices maintaining the same strength as cases at smaller $\theta_{0}$. The explanation could be traced back to the fact that when $2^{u}$, $2^{d}$ becomes less energetic, $1^{u}$, $1^{d}$ detach slightly earlier and as a consequence they move upwards (downwards) in downstroke (upstroke) switching their role from detrimental to beneficial in terms of propulsive performance averaged over the entire period.

### 4.3. Strouhal Number and Heave Amplitude

In Figure 4a, the combined effects of Strouhal number and heave amplitude are portrayed, keeping fixed $\theta_{0}=15^{\circ }$ and $\psi =90^{\circ }$. Note that by maintaining constant pitch amplitude and phase shift, the configurations characterized by the same St number produce the same $\alpha_{max}$, as one can write

(20) $$\alpha \left(t\right)=\theta_{0}\sin\left(\frac{2\pi t}{T}\right)+\arctan\left(\pi St\cos\left(\frac{2\pi t}{T}+\psi \right)\right)\;,$$

with an effect of the heave amplitude corresponding just to a direct modulation of the motion period $T=1/f=2h_{0}T_{flow}/\left(cSt\right)$, with $T_{flow}=c/U_{\infty }$ being the time scale of the flow. Equation (20) hence shows that, for a fixed St, the term $T\dot{\alpha }$ does not depend on the heave amplitude, see also Figure 4b, and in particular as $h_{0}$ is increased $\dot{\alpha }$ decreases and vice versa.

The maximum efficiency manifests over a neighborhood centered in St=0.2, with a peak value above 85% for $h_{0}=3c$ at St=0.1935. The fact that for all heave amplitudes $\eta_{p_{max}}$ occurs in such range is in line with what was documented in [5,15,43] and reflects the assertion advanced in [14,16,44,45], namely that most fishes swim following this flapping frequency, which corresponds to the most efficient from a propulsive standpoint. The mean thrust coefficient monotonically increase along with St due to the continuous increment of lift owed to the onset of dynamic stall effects, as already mentioned in Section 4.1.

**Figure 11:**
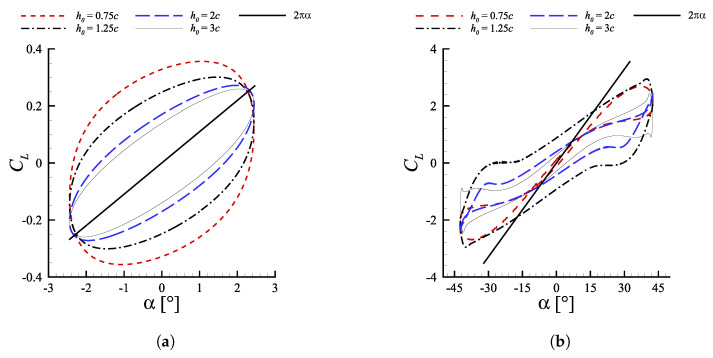
$\left(C_{L},\;\alpha \right)$ diagrams at $\psi =90^{\circ }$ and $\theta_{0}=15^{\circ }$ for varying $h_{0}$ at (**a**) St=0.1 and (**b**) St=0.5.

Another distinctive feature, detectable in Figure 4a, is the significantly different effect of heave amplitudes on the propulsion performance at small and large St. As far as $\eta_{p}$ is concerned, the cases with $h_{0}=3c$, which have the highest $h^{*}$, present the best efficiency at low St. After such optimum, $\eta_{p}$ decreases very fast moving towards higher Strouhal numbers, with the configuration $h_{0}=3c$ becoming the least efficient after St≈0.25. These findings confirm what reported by Akoz et al. [17], according to which heave-dominated motions maximize their efficiency at low Strouhal numbers. On the other hand, the configuration $h_{0}=0.75c$, generating the least optimal efficiency among the ones that have been investigated, exhibits the most subtle $\eta_{p}$ decrease at high Strouhal numbers, demonstrating to be the most efficient one when high angles of attack are involved. An almost identical trend might be observed in the ${\bar{C}}_{T}$ curves. As a matter of fact when the Strouhal number is low the mean thrust coefficient is maximum for $h_{0}=3c$, instead at high St the propelling force is optimal for $h_{0}=0.75c$. An helpful insight on such dynamics might be extracted from the visualization of the phase diagrams reported in Figure 11. Therein we consider the lift coefficient defined as

(21) $$C_{L}=\frac{L}{1/2\;\rho \;b\;c\;U_{eff}^{2}}\;,$$

against the values assumed by α during one motion period. Two cases, in particular, are analyzed as they show an opposite behavior with respect to the heave amplitude: (i) St=0.1 and (ii) St=0.5.

At St=0.1 a motion regime for which $\alpha_{max}\;<\;3^{\circ }$ is established. Due to low angles of attack, the effects of stall delay coming from a fast dynamics are not triggered, with the latter seeming to mainly induce strong energy-loss features. This is reflected in Figure 11a by the presence of a distinguishable reduction of the hysteresis cycle as $h_{0}$ is increased. Such progressive collapse of the $C_{L}\left(\alpha \right)$ curve appears to produce an alignment with the 2πα law, characteristic of steady, inviscid aerodynamics.

On the other hand, as the Strouhal increases a parallel increment of the $\alpha_{max}$ experienced by the profile during its cycle occurs. At St=0.5, Figure 11b, a value $\alpha_{max}=42.52^{\circ }$ is reached as the profile pass through the zero vertical position. For such values of angle of attack, well above the static stall, the trend-inversion in the effects of increasing heave amplitude on the propulsion performances does not come as a surprise. Indeed, the beneficial effects of a fast dynamics, allowing for a separation delay and a continuous lift production result to be strongly mitigated when increasing $h_{0}$, due to the associated decreasing effect on motion frequency. Referring again to Figure 11b, for the configurations with $h_{0}<2c$ the dynamic contribution coming from large $\dot{\alpha }$ enables to keep generating lift up to $\alpha_{max}$, reaching peak values after crossing the position corresponding to h=0, i.e., as the profile decelerates towards its maximum stroke displacement. Conversely, when $h_{0}\geq 2c$ the reduction of $\dot{\alpha }$ causes a decrease in the maximum sustainable angle of attack before occurrence of deep stall and thrust collapse. In particular, for $h_{0}=3c$ the lift coefficient experiences a sudden drop after a localized peak at $\alpha \approx 41^{\circ }$. Such behavior is consistent with the onset of a deep stall, featured by the presence of a chord-sized LEV arising from leading edge separation, before reaching $\alpha_{max}$.

Figure 7 includes the vorticity fields at St=0.5 for the values of $h_{0}$ reported above. As $h_{0}\leq 1.25c$, Figure 7a,b, both captured during upstroke, the frequency variation has a clear manifestation in the modification of the pattern of the shed eddies. In particular, for $h_{0}=0.75c$ the high motion frequency, inducing large $\dot{\alpha }$ values, avoid significant leading edge separation as the profile travels through $\alpha_{max}$ while for $h_{0}=1.25c$ a distinct separated region of positive vorticity is clearly distinguishable. When $h_{0}\geq 2c$, Figure 7c,d, a chord-sized LEV detaches from the solid boundary as the angle of attack approaches its maximum in the cycle, with the profile entering in deep stall. For $h_{0}=3c$, Figure 7d, a negative vorticity region close to the profile identifies the further shedding of a large trailing edge vortex (TEV). It might be conjectured, as also reported in [39], that as the formed LEV detaches and starts being advected downstream, i.e., as the deep stall comes into being, the sudden change of pressure distribution around the profile forces the roll-up of the opposite vorticity boundary layer, creating the above mentioned TEV that, therefore, can be considered as a further sign of deep stall onset.

In conclusion, independently on the St regime at which the profile operates, it would seem that efficiency-driven propulsion solutions should mainly focus on kinematic configurations ensuring minimal, if not absent, leading edge separation. At low Strouhal this is guaranteed, provided an adequate choice of phase shift is performed, see Section 4.4, by the low angles of attack that are produced, whereas when dealing with fast dynamics regime an effective thrust production, even though sub-optimal, requires low heave.

### 4.4. Phase Shift

The phase offset between pitch and heave sinusoids establishes whether the leading edge leads the trailing edge or vice versa. Figure 5a shows the propulsion characteristics as function of the flapping phase angle. In this case ψ was varied between $45^{\circ }$ and $120^{\circ }$ with a constant step of $15^{\circ }$ and between $150^{\circ }$ and $180^{\circ }$ with a step of $10^{\circ }$, keeping the remaining parameters fixed at St=0.1935, $\theta_{0}=15^{\circ }$ and $h_{0}=c$. This choice was made in order to include both the interval where efficiency have been reported as the highest [35,36] and the regions where $\eta_{p}$ undergoes a considerable degradation.

**Figure 12:**
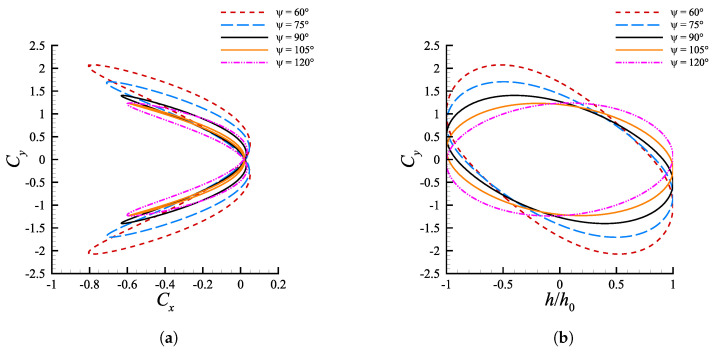
(**a**) $\left(C_{y},C_{x}\right)$ and (**b**) $\left(C_{y},h/h_{0}\right)$ diagrams at varying phase shift for cases with St=0.1935 and $\theta_{0}=15^{\circ }$. The curves in the $\left(C_{y},h/h_{0}\right)$ space orbit counterclockwise.

According to Van Buren et al. [36] the phase shift is a crucial factor, and this is corroborated by Figure 5, where both $\eta_{p}$ and ${\bar{C}}_{T}$ are subjected to large variations, in an interval that covers less than one-half of the entire spectrum of phases. The mean thrust tends to increase moving away from $\psi =90^{\circ }$ due to the increase of the angle of attack, which increments the lift, nevertheless drag increases even more and causes $\eta_{p}$ to diminish. Once $\psi >150^{\circ }$ then $\alpha_{max}$ becomes too high and ${\bar{C}}_{T}$ collapses, producing in turn a drop of the efficiency. The cause is that $\dot{\alpha }$ becomes insufficient to avoid deep stall at angles of attack greater than $30^{\circ }$. The phase shift, in fact, seems to have a rather bland effect on $\dot{\alpha }$ (see Figure 5b) and it does not allow to obtain values high enough to counteract the simultaneous growth of α. The efficiency is maximized at $105^{\circ }\leq \psi \leq 120^{\circ }$, in between the results outlined by Van Buren et al. [36] and what is predicted by linear theory, for which $\eta_{p_{max}}$ verifies at $\psi =90^{\circ }$ and $\psi \approx 135^{\circ }$, respectively.The diverse behavior of efficiency and mean thrust coefficient at phase angles greater or lower than $90^{\circ }$ can be investigated looking at Figure 12. Among the simulated cases, the efficiency is optimal at $\psi =105^{\circ }$ because it allows to change the angle of attack in such a way to boost $C_{y}$ in the first half of the upstroke and downstroke phases and diminish it in the second halves, as showed in Figure 12b. This reflects in a minimization of the hysteresis in the $\left(C_{y},C_{x}\right)$ space (Figure 12a), which is directly linked to $\eta_{p}$ as pointed out in Section 4.3. Aside from the hysteresis one can also note in Figure 12a that for $\psi <90^{\circ }$ the thrust coefficient reaches higher values throughout the oscillating cycle (we recall that $C_{T}=-C_{x}$), whereas for $\psi >90^{\circ }$ the orbits span between smaller values.

**Figure 13:**
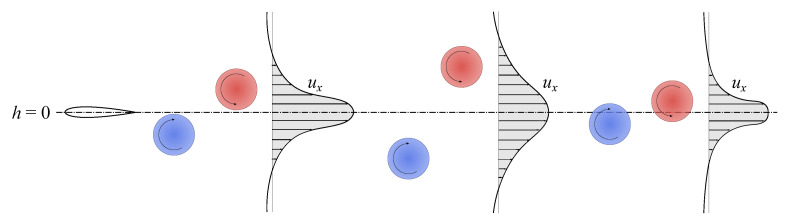
Sketch of the streamwise velocity profiles for counter-rotating vortices travelling at different distances from the neutral axis.

Figure 8 displays how the vorticity field is affected by the phase shift variation. In the interval considered, the flows past the foil are almost identical when $\psi \in [75^{\circ },120^{\circ }]$ and this finds confirmation in their very similar propulsive performance. The wake topology, in particular, tends to a 2S reverse von Kármán. The only visible difference among the various cases is the width of the wake, which decreases as ψ increases. This could justify the highest efficiency at $\psi \approx 105^{\circ }$ through the observation that the single vortices shed each half cycle are closer to the neutral axis. Indeed, as long as the counterclockwise vortices are above h=0 and the clockwise vortices are below it, the propelling action is more effective if the eddies are close to the neutral position, since the velocity distributes as a concentrated jet. A schematic representation of this mechanism is illustrated in Figure 13. Outside of the interval $\left[75^{\circ },120^{\circ }\right]$ the wake becomes 2P, although starting from $\psi \geq 170^{\circ }$ it tends to go back to a 2S pattern. Specifically, the change in slope of $\eta_{p}$ at $\psi \approx 160^{\circ }$ in Figure 5 may be associated to the detrimental vortices $1^{u},1^{d}$ which move upward and vertically align with the eddies $2^{u},2^{d}$. Their alignment causes their contributions to forward motion to cancel out, leading to little neat thrust. Once $\psi \geq 170^{\circ }$ the vortices $1^{u},1^{d}$ almost vanish, however $2^{u},2^{d}$ are squeezed towards the centerline and their contribution starts to cancel out, as discussed right before.

## 5. Conclusions

Several simulations involving the dynamics of a flapping NACA0015 profile were performed on top a discontinuous Galerkin solver, where the high-order numerical framework was exploited to ensure the resolution independence of the output data.

A wide range of kinematic configurations was presented, involving multiple heaving amplitudes, Strouhal numbers, pitch and phase angles, so to replicate the typical operational conditions of BCF swimmers. A detailed analysis of the problem sensitivity upon such variables was performed, and original observations concerning the relevance of dynamic stall and phase shift effects were advanced.

The results highlighted that, when employing thrusters with an imposed kinematics that makes use of circulatory-based propulsion mechanisms, the most relevant features influencing the propulsive performance appear to be the angle of attack and its rate of change. Upon inspection of the maximum angles of attack in the space of the tested configurations, the presence of a global optimum from an efficiency standpoint clearly emerged, located in the range $15^{\circ }<\alpha_{max}<20^{\circ }$. The display of a localized peak at such values well above static stall, and the retaining of considerably high efficiency values for even larger $\alpha_{max}$, might be traced back to a leading edge separation delay imposed by the unsteady motion effects, associated to the sinusoidal $\dot{\alpha }$. Furthermore, considering simple balance equations it was showed, for circulatory-dominated propulsion, the necessity of constraining the operational conditions within a regime ensuring high lift-to-drag ratios, so to guarantee high propulsive efficiency.

As far as pitch angle is concerned, the explored configurations manifested an optimal efficiency for $15^{\circ }<\theta_{0}<20^{\circ }$, corresponding to the interval in which the lift-to-drag ratio of the profile is maximized. Furthermore, the inspection of the spanwise vorticity fields underlined the influence of the pitch amplitude on the vorticity pattern arising behind the profile. Specifically, vortices seems to be formed at two distinct separation stages, namely once the maximum instantaneous angle of attack is reached and when rate of change of α assumes the highest value.

Concerning the effects of Strouhal number and heave amplitude, it emerged once again the key role played by the dynamics of α. For fixed $\theta_{0}$ and ψ, a given Strouhal number directly sets $\alpha_{max}$, while $h_{0}$ directly modulates the frequency of the motion cycle. Optimal efficiency was found in the interval 0.2<St<0.3, while mean thrust coefficient increases monotonically up to St=0.7, in line with the majority of the works published on the subject. For values of $\alpha_{max}$ well above the static stall condition, large heave amplitudes, i.e. high frequencies, emerged as a feasible strategy to get very high efficiency. Such condition, indeed, results in an increase of $\dot{\alpha }$ which allows to postpone leading edge separation and maintain significant lift production at large angles of attack. On the other hand, when the maximum angle of attack reached during the oscillating period is smaller than the static stall value, an efficiency improvement was observed for narrow heave amplitudes. For such cases the low frequency also brought to a decrease of the hysteresis in the $\left(C_{y},C_{x}\right)$ plane.

A similar behavior in terms of hysteresis reduction was also detected when examining the performance sensitivity to the phase shift. From a dynamic viewpoint, the reduction of the loads hysteresis cycle appears to have the function of compensating the dynamic effects owed to the profile motion. An adequate choice of ψ, in particular, allows for a faster inversion of the lift sign in the transition between upstroke and downstroke, limiting the portion of the cycle where the profile might have a detrimental propulsive behavior. The results showed an optimal efficiency for $105^{\circ }<\psi <120^{\circ }$, which in literature is typically identified at $\psi =90^{\circ }$.

## Figures and Tables

**Table 1:** Propulsive efficiency and mean thrust coefficient of the whole set of kinematic configurations.

$\theta_{0}$ [${}^{\circ }$]	$h_{0}/c$	ψ [${}^{\circ }$]	St	$\alpha_{\max}$ [${}^{\circ }$]	$h^{*}$	$\eta_{p}$ [%]	${\bar{C}}_{T}$
1	1	90	0.1935	30.30	1.00	55.3	0.442
5	1	90	0.1935	26.30	0.99	67.5	0.439
10	1	90	0.1935	21.30	0.97	72.7	0.373
15	1	90	0.1935	16.30	0.94	75.4	0.295
20	1	90	0.1935	11.30	0.90	75.4	0.205
25	1	90	0.1935	6.30	0.86	68.3	0.103
15	0.75	90	0.1	2.44	0.90	23.6	0.006
15	0.75	90	0.2	17.14	0.90	70.4	0.288
15	0.75	90	0.3	28.30	0.90	61.4	0.756
15	0.75	90	0.5	42.52	0.90	44.0	2.236
15	0.75	90	0.7	50.55	0.90	25.6	3.806
15	1	90	0.1	2.44	0.94	35.8	0.010
15	1	90	0.1935	16.30	0.94	75.4	0.295
15	1	90	0.3	28.30	0.94	65.5	0.833
15	1	90	0.5	42.52	0.94	42.1	2.134
15	1	90	0.7	50.55	0.94	26.9	3.697
15	1.25	90	0.1	2.44	0.96	43.9	0.013
15	1.25	90	0.2	17.14	0.96	78.2	0.350
15	1.25	90	0.3	28.30	0.96	67.8	0.902
15	1.25	90	0.5	42.52	0.96	37.3	1.969
15	1.25	90	0.7	50.55	0.96	24.2	3.393
15	1.5	90	0.1	2.44	0.97	48.5	0.016
15	1.5	90	0.2	17.14	0.97	80.2	0.374
15	1.5	90	0.3	28.30	0.97	66.9	0.957
15	1.5	90	0.5	42.52	0.97	33.6	1.834
15	1.5	90	0.7	50.55	0.97	22.0	3.164
15	1.75	90	0.1	2.44	0.98	52.6	0.018
15	1.75	90	0.2	17.14	0.98	81.9	0.394
15	1.75	90	0.3	28.30	0.98	61.5	0.933
15	1.75	90	0.5	42.52	0.98	29.9	1.730
15	1.75	90	0.7	50.55	0.98	20.3	2.913
15	2	90	0.1	2.44	0.99	54.7	0.019
15	2	90	0.2	17.14	0.99	83.2	0.411
15	2	90	0.3	28.30	0.99	56.4	0.876
15	2	90	0.5	42.52	0.99	25.1	1.450
15	2	90	0.7	50.55	0.99	15.9	1.593
15	2.5	90	0.1	2.44	0.99	57.6	0.021
15	2.5	90	0.2	17.14	0.99	84.9	0.435
15	2.5	90	0.3	28.30	0.99	49.1	0.807
15	2.5	90	0.5	42.52	0.99	23.3	1.228
15	2.5	90	0.7	50.55	0.99	16.0	1.521
15	3	90	0.1	2.44	0.99	59.2	0.023
15	3	90	0.2	17.14	0.99	85.9	0.450
15	3	90	0.3	28.30	0.99	46.5	0.767
15	3	90	0.5	42.52	0.99	23.0	1.173
15	3	90	0.7	50.55	0.99	15.9	1.554
15	1	45	0.1935	23.90	1.16	63.8	0.416
15	1	60	0.1935	20.28	1.08	68.0	0.363
15	1	75	0.1935	17.45	1.00	71.6	0.322
15	1	90	0.1935	16.30	0.94	75.4	0.295
15	1	105	0.1935	17.45	0.90	77.7	0.281
15	1	120	0.1935	20.28	0.86	77.6	0.280
15	1	150	0.1935	27.80	0.82	68.7	0.296
15	1	160	0.1935	30.39	0.81	61.4	0.297
15	1	170	0.1935	32.90	0.81	46.8	0.285
15	1	180	0.1935	35.27	0.80	25.2	0.175

## Data Availability

The data presented in this study are available upon request to the corresponding author.

## Supplementary Materials

The following supporting information can be downloaded at: https://www.mdpi.com/article/10.3390/biomimetics8020135/s1, Video S1: Time evolution of the vorticity field in Figure 6a. Video S2: Time evolution of the vorticity field in Figure 6b. Video S3: Time evolution of the vorticity field in Figure 6d. Video S4: Time evolution of the vorticity field in Figure 7c.

## Abbreviations

The following abbreviations are used in this manuscript:

AUV	Autonomous underwater vehicle
BCF	Body and caudal fin
CFD	Computational fluid dynamics
DG	Discontinuous Galerkin
DSV	Dynamic stall vortex
GMRES	Generalized minimal residual method
LEV	Leading edge vortex
L/D	Lift-to-drag
TEV	Trailing edge vortex
URANS	Unsteady Reynolds-Averaged Navier-Stokes
